# Holistic Approach to the Uncertainty in Shelf Life Prediction of Frozen Foods at Dynamic Cold Chain Conditions

**DOI:** 10.3390/foods9060714

**Published:** 2020-06-02

**Authors:** Maria Giannakourou, Petros Taoukis

**Affiliations:** 1 Department of Food Science and Technology, University of West Attica, 12243 Athens, Greece; 2 Laboratory of Food Chemistry and Technology, School of Chemical Engineering, National Technical University of Athens, 15780 Athens, Greece; taoukis@chemeng.ntua.gr

**Keywords:** cold chain, frozen foods, shelf life modeling, uncertainty, variability, joint confidence intervals, Monte Carlo

## Abstract

Systematic kinetic modeling is required to predict frozen systems behavior in cold dynamic conditions. A one-step procedure, where all data are used simultaneously in a non-linear algorithm, is implemented to estimate the kinetic parameters of both primary and secondary models. Compared to the traditional two-step methodology, more precise estimates are obtained, and the calculated parameter uncertainty can be introduced in realistic shelf life predictions, as a tool for cold chain optimization. Additionally, significant variability of the real distribution/storage conditions is recorded, and must be also incorporated in a kinetic prediction scheme. The applicability of the approach is theoretically demonstrated in an analysis of data on frozen green peas Vitamin C content, for the calculation of joint confidence intervals of kinetic parameters. A stochastic algorithm is implemented, through a double Monte Carlo scheme incorporating the temperature variability during distribution, drawn from cold chain databases. Assuming a distribution scenario of 130 days in the cold chain, 93 ± 110 days remaining shelf life was predicted compared to 180 days assumed based on the use by date. Overall, through the theoretical case study investigated, the uncertainty of models’ parameters and cold chain dynamics were incorporated into shelf life assessment, leading to more realistic predictions.

## 1. Introduction

One of the principal goals in food science and industrial practice is to develop ways to improve quality of foods, by means of controlling chemical, physical and microbiological changes during processing and storage [1]. In most cases, food quality is gradually deteriorating, as food matrices are physicochemically and biologically active systems [2]. Increased interest is focused on deriving kinetic information for different food products, so as to calculate change rates, and thus be able to estimate their shelf life. In order to be able to reliably assess product quality during processing and storage, appropriate mathematical equations and kinetic parameters are necessary. Further to the mean estimate of such parameters, complementary statistical evaluation methodology should also be employed, to allow for reliable assessment of the uncertainty of calculations in the conditions of the cold chain [3].

When reviewing literature on frozen food quality deterioration, most available experimental data are provided at a reference temperature (e.g., −18 °C) [4] and single measurements at selected, limited time points (e.g., after 6 and 12 months of storage) for different quality parameters of the food system [5,6,7,8]. Although practical for comparisons between different freezing processes, this approach cannot be further used for a meaningful quantitative projection of the quality status at other time-temperature regimes and most importantly at the dynamically variable conditions of the different stages of the frozen distribution chain. A thorough experimental design and measurement of quality and methodical application of kinetic principles leading to the development and validation of mathematical models at a number of conditions covering the entire temperature domain of practical application is required for accurate predictions on dynamic systems [9,10,11].

While not overlooking the complexity of phenomena including freeze concentration, glass transition, ice crystallization, etc. [12,13] that influence the kinetics of quality loss during storage of frozen matrices, in the majority of published works the “apparent kinetics” methodological approach is used. This includes two main successive calculations. A primary kinetic model is developed via best statistical fit to describe the selected quality parameter change as a function of processing time or post-processing storage/distribution and a secondary model that reflects the effect of processing factors and/or environmental conditions on primary model’s parameters [14]. Once validated, these mathematical formulae can be a practical tool to predict post-processing quality status at any stage of food storage.

Alternatively, the model parameters can be determined in a single step considering the same isothermal dataset as a whole, by incorporating the secondary model equations into the primary model and performing a non-linear regression [15]. Current publications have proposed and employed one-step kinetic analysis [1,16,17,18,19,20,21]. Such approach circumvents the need for statistical estimation of intermediate parameters by employing all the experimental data in a single non-linear algorithm [22], with higher number of degrees of freedom, leading to more precise parameter calculation. The drawback could be in the selection of the correct optimization algorithm and fitting criterion [1]. Authors in [23], using a hypothetical isothermal inactivation data set, and applying first order kinetics, studied the effect of the data regression technique for three least squares regression methods, based on the Arrhenius model as the secondary model. It was concluded that non-linear least squares regression leads to unbiased and precise estimation of the Arrhenius parameters, without performing unnecessary intermediate calculations. In any case, this approach requires more sophisticated computational tools and possibly the need to perform complicated iterative algorithms, a complexity that makes many researchers prefer the much more convenient two-steps procedure.

Regardless of the applied methodology (2-step and 1-step), an often occurring problem in practical applications is that the overall error of the primary and secondary kinetic equations is not accounted for. By using the mean parameter values, the single estimates resulting from the models may encompass serious uncertainty especially under variable and fluctuating conditions [24].

When reviewing frozen food kinetic studies, one could observe that there are weak points, mainly related to the uncertainty of the model parameters based on the determination of the exact confidence intervals [25]. Even if calculated, these ±95% confidence intervals of model parameters are not taken into account when estimating the quality retention, at any given conditions. This deterministic approach to predict the remaining shelf life is useful as a practical tool but caution should be applied as far as its real accuracy. A systematic approach fully accounting for the statistical uncertainty of model parameters, and allowing for increased reliability in shelf life estimations could be based on Monte Carlo simulation techniques. Based on the employment of such tools, the stochastic variability and uncertainty associated with various quality attributes of different food matrices [16,18,26,27,28,29] has been successfully described in current scientific publications.

Going a step further to this kinetic approach, accounting for the ±95% confidence intervals of the parameters of the secondary models would require taking into account the interrelation between them, usually considered as ‘independent’, parameters. This correlation between the kinetic parameters, e.g., between the pre-exponential constant and the activation energy, E_a_, of the Arrhenius equation, means that the confidence interval of one parameter depends on the value of the other parameter [30]. Joint confidence contours can be used to account for this statistical interdependence. Such plots provide information on the combinations of model parameter values, that are encompassed by the joint confidence ellipsoid. This would allow for the exemption of combinations that fall outside the ellipsoid that denotes the joint confidence boundary [31,32,33]. In recent literature, there are scarce studies that have estimated and plotted the joint confidence intervals of kinetic parameters [25,31,34,35,36,37,38]. Such approach would be particularly appropriate in order to account for the real uncertainty of model parameters, and thus proceed to realistic and reliable shelf life estimations, using Monte Carlo simulation. Although Monte Carlo techniques have been applied in recent literature for the probabilistic assessment of stochastic variability and uncertainty associated with various quality attributes of different food systems [16,18,26,27,28,29], the statistical interrelation of the values applied in the iterative simulation process has not been considered.

Besides overlooking model parameter uncertainty (best expressed by the ±95% joint confidence plots), another problematic practice in shelf life determinations and predictions refers to assumed fixed temperature conditions at different stages of the cold chain, despite the well-known deviations that occur. Therefore, it would be important to also account for the real variability of cold chain conditions.

The objective of this work was to holistically approach shelf life calculations through a double Monte Carlo technique applied to frozen food kinetics data, taking into account both kinetic parameter uncertainty and temperature variability at all stages of the actual cold chain. This will be demonstrated via case study computations encompassing the effect of different scenarios of cold chain control. The ultimate goal of this holistic approach is to obtain realistic shelf life predictions, examine the main factors affecting shelf life calculations through a sensitivity analysis and provide a tool for effective management and optimization of frozen food cold chain.

## 2. Materials and Methods

### 2.1. Basic Principles

According to the two-steps kinetic approach to model food quality degradation, the most representative quality attributes are carefully selected, their change is systematically assessed over time under isothermal storage conditions and an appropriate mathematical equation (primary model) is applied in order to describe this change. Subsequently, a secondary model is chosen to depict the effect of the most important factors (e.g., temperature), on the rate of changes. An alternative approach involves the implementation of a 1-step analysis, through a non linear fitting algorithm that uses an integrated unique equation, deriving more precise estimations [39].

Primary models describe the rate of loss of one or more quality factors, such as a nutrient or a pigment or the rate of the production of an undesirable compound, such as an off-flavor or discoloration (Equation (1)):

(1) $$r_{A}=\pm \frac{d\left(A\right)}{\mathrm{dt}}=\;k\cdot {\left(A\right)}^{n}$$

where the quality index (A), that may represent a chemical, physical, microbiological or sensory attribute, is chosen to most representatively describe the quality degradation of the food system studied. The constant k is the reaction rate constant and n is the apparent order of the respective reactions. In order to calculate the values of k and n of Equation (1), the change of the experimentally measured values of (A) should be appropriately fitted with time; a mathematical function, frequently called “the Quality function” (Q(A)) vs. time is then derived [40], that allows for quality quantification. This function incorporates the dependency of quality change on several intrinsic and extrinsic factors, through their effect on the reaction rate constant, k (k = f (C_i_, E_j_), with C_i_ describing factors related to food composition and E_j_ representing different environmental factors [41,42].

As far as secondary models are concerned, the Arrhenius equation (Equation (2)) is mostly applied to describe temperature dependence of quality changes of frozen foods [25]. Nonetheless, there have been serious arguments highlighting its restrictions in describing frozen matrix behavior in the temperature range near the glass transition temperature [43,44,45]. In [46,47], the pros and cons of the alternative secondary models often used for frozen foods, namely Arrhenius and WLF, are presented in detail, underlying their cautious applicability at the lower boundary of the temperature range investigated, since their logarithmic functions render them sensitive to rates.

(2) $$k\;={\;k}_{\mathrm{ref}}\left[\frac{-E_{a}}{R}\left(\frac{1}{T}-\frac{1}{T_{\mathrm{ref}}}\right)\right]$$

where k_ref_ is the rate constant at the reference temperature T_ref_ (K), R: the universal gas constant and E_a_: the activation energy (J/mol or cal/mol).

In the case of frozen foods, the time of experimental measurements for a full kinetic study is often times impracticably long. In order to expedite the experimental procedure, without jeopardizing the accuracy of the results, the two-step kinetic methodology is implemented via the Accelerated Shelf Life Testing (ASLT) approach [48,49]. The value of the Q(A_t_) quality function at time t, defined by Equation (1) in the case of isothermal conditions, is calculated, with T(t) describing the change of temperature as a function of time:

(3) $$Q\left(A_{t}\right)=\overset{t_{\mathrm{tot}}}{\underset{0}{\int }}k\left[T\left(t\right)\right]\cdot \mathrm{dt}\;={\;k}_{\mathrm{eff}}\cdot t_{\mathrm{tot}}$$

where k_eff_ is the value of the rate of the quality loss reaction at the effective temperature T_eff_. The T_eff_ term represents the constant temperature that results in the same quality value as the variable temperature function over the same time period, which equals t_tot_.

If the T(t) distribution is discretized in small time increments t_i_ of constant temperature T_i_ (with $\sum^{\;}t_{i}={\;t}_{\mathrm{tot}}$), and applying the Arrhenius equation as the secondary model, then Equation (3) can be alternatively written as Equation (4):

(4) $$k_{\mathrm{ref}}\cdot \underset{i}{\sum }\left[\exp\left[-\frac{E_{a}}{R}\cdot \left(\frac{1}{T_{i}}-\frac{1}{T_{\mathrm{ref}}}\right)\right]\cdot t_{i}\right]={\;k}_{\mathrm{eff}}\cdot t_{\mathrm{tot}}$$

From Equation (4), the value of k_eff_ can be estimated and subsequently, from the Arrhenius model, the effective temperature T_eff_ can be calculated.

### 2.2. Model Development and Determination of the Uncertainty of Kinetic Parameters

As a case study to test the proposed methodology, data from literature on the shelf life of frozen green peas was used [50], where Vitamin C loss was systematically measured at five constant temperatures. A first-order reaction order (primary model) was established for this chemical reaction (Equation (5)) and the Arrhenius equation was applied to efficiently quantify the temperature effect (secondary model), (Equation (2)):

(5) $$C\;={\;C}_{0}e^{-k_{\mathrm{vitC}}t}$$

where C_0_ the initial Vitamin C concentration (mg/100 g of food), k_VitC_ is the reaction rate of the Vitamin C oxidation at a fixed temperature and at Equation (2) T_ref_ for frozen foods equals to −20 °C.

If a one step analysis is performed, a single equation (Equation (6)) that integrates both primary and secondary models (Equations (2) and (5)) is used and a non-linear regression is applied (SYSTAT 8.0).

(6) $$\frac{C}{C_{0}}=\;\exp\left(\left(-k_{\mathrm{ref}}\cdot \exp\left(\frac{-E_{a}}{R}\cdot \left(\frac{1}{T}-\frac{1}{T_{\mathrm{ref}}}\right)\right)\right)\cdot t\right)$$

then, one can estimate the mean value, as well as the 95% Confidence Intervals (C.I.) of the kinetic parameters, namely E_a_ (in kJ/mol) and k_ref_ (at −20 °C, in days^−1^).

In order to estimate the Confidence Intervals of Equation (6) one needs to calculate the first derivative of the dependent variables. A model is linear in a parameter if the derivative with respect to that parameter is not a function of that parameter [25]. In the studied case, our model is non linear (since both $\frac{\vartheta \left(\frac{C}{C_{0}}\right)}{\vartheta k_{\mathrm{ref}}}=\;f\left(k_{\mathrm{ref}}\right)\;\mathrm{and}\;\frac{\vartheta \left(\frac{C}{C_{0}}\right)}{\vartheta E_{a}}=\;f\left(E_{a}\right)$); then the parameter cannot be solved for directly, but only through a non linear regression. The estimation of such non symmetric confidence intervals is not straightforward and the extent of their asymmetry depends on the nonlinearity of the function and the number of data [38]. As stressed out in [30], it is essential to describe the methodology used for confidence intervals calculation, in order to be able to interpret their meaning. In this work, MATLAB^®^ was used and appropriate code was written, in order to derive the asymptotic standard errors (SE), calculated by Equation (7) based on the t-parameter for a confidence level (±SE_∙t(1–0.5a),n)_ ([1,51]).

(7) $$\left[\begin{array}{cc} SE_{k_{ref}}^{2} & SE_{k_{ref},E_{a}}\\ SE_{k_{ref},E_{a}} & SE_{E_{a}}^{2} \end{array}\right]=\left(J^{T}\cdot J\right)\cdot \frac{SSE}{n-p}$$

where J is the Jacobian matrix (Equation (8)), which is calculated by the partial derivatives of the model output with respect to the model parameters estimated at each measurement point, p is the number of estimated parameters (p = 2, in this work), n is the number of observations, the superscript T denotes the matrix/vector transpose operator and SSE represents the sum of standard errors.

(8) $$J=\left[\begin{array}{cc} \frac{\vartheta Y_{1}}{\vartheta k_{ref}} & \frac{\vartheta Y_{1}}{\vartheta E_{a}}\\ \vdots & \vdots \\ \frac{\vartheta Y_{n}}{\vartheta k_{ref}} & \frac{\vartheta Y_{n}}{\vartheta E_{a}} \end{array}\right]$$

where $Y=\frac{C}{C_{o}}$

The 95% C.I. are calculated based on Equations (7) and (8) [38], and the relative MATLAB command nlparci([kref,ea],res,j), where the res term, representing the Residuals, is calculated using Equation (9) at each experimental point, i:

(9) $$Residual_{i}={\left(\frac{C}{C_{o}}\right)}_{obS,i}-{\left(\frac{C}{C_{o}}\right)}_{pred,i}$$

The confidence level selected actually expresses the probability that the confidence interval produced will contain the true parameter value. For example, a 95% confidence interval covers 95% of the normal curve and the probability of obtaining a value outside of this area is less than 5%. Therefore, it is assumed that the Arrhenius parameters E_a_ and k_ref_ can be described by a normal distribution curve, rather than a single value. Implementing this assumption, the variability calculated by the model of Equation (6) is incorporated within calculations of the shelf life of frozen green peas.

The next step in this analysis of the 1-step non-linear regression on the isothermal data, is to investigate the covariance of the simultaneously estimated parameters, by constructing the, joint confidence regions, according to the expression [52] (Equation (10)):

(10) $$SSE\leq SSE\left(\theta \right)\left\{1+\frac{p}{n-p}F\left(p,n-p,1-a\right)\right\}$$

where SSE(θ) is the least sum of squared differences, at optimal parameter values, and F is the upper 1 − a quantile for an F-distribution with p and n − p degrees of freedom.

All combinations of kinetic parameters with sum of squares less than or equal to the calculated SSE(θ) values will be inside the joint confidence region. The confidence regions obtained with this method (Equation (9), called likelihood confidence regions, can be disjoint and unbounded [32]. All of our calculations were based on the iterative method of [53], which is described in [38].

### 2.3. Determination of the Variability of Temperature Conditions in the Cold Chain

**Figure 1:**
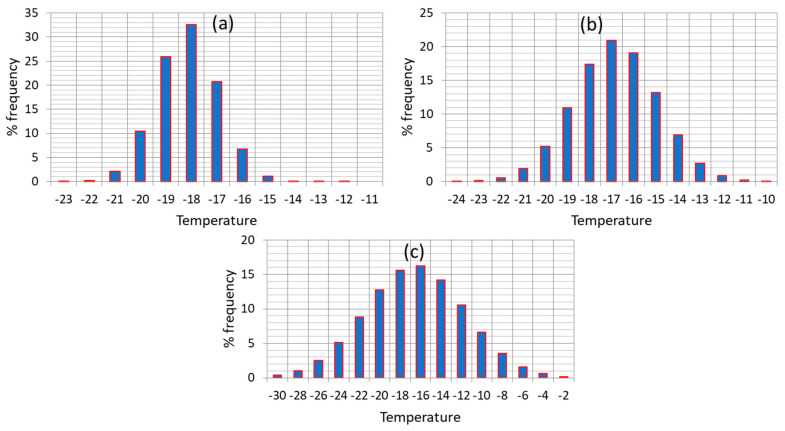
Temperature distribution during (**a**) production/distribution warehouse, (**b**) retail display and (**c**) domestic storage for frozen foods handling (FRISBEE database).

When addressing the issue of shelf life determination, most studies on frozen foods refer to an average temperature throughout product handling in the cold chain. This oversimplistic assumption, besides being false, could possibly lead to unrealistic predictions of quality change and remaining shelf life, at any point of the cold chain. Consequently, it is crucial to account for the real temperature history of the product in the cold chain (including fluctuations and abusive conditions), in order to be able to predict in a more accurate way quality changes, at any point of the cold chain (at storage or distribution). Based on field studies of the real handling of frozen foods in the cold chain [54,55,56], a variable temperature environment was recorded, which often included stages of abusive storage or transport/transfer conditions. In order to estimate in a realistic way the loss of quality parameters at each stage, and predict accurately the remaining shelf life at the end of the cold chain, it is important to fully account for the effect of temperature history by incorporating the fluctuations occurring in the real cold, post-processing chain, and the conditions’ variability within the model prediction algorithm. A very useful tool that provides plenty of data concerning frozen foods handling, in all distinct stages of storage/transport/distribution is the FRISBEE project cold chain database (www.frisbee-project.eu/coldchaindb, [55]) 1841 records of production warehouse, 53 records of distribution warehouse, 636 records of retail display and 354 records of domestic freezers were retrieved and statistically analyzed, in order to incorporate temperature variability within shelf life predictions, at any point of the cold chain. In Figure 1, temperature distribution of the three main stages of frozen food handling is depicted, based on the aforementioned statistical treatment of the database records.

### 2.4. Shelf Life Assessment and Uncertainty Determination

**Figure 2:**
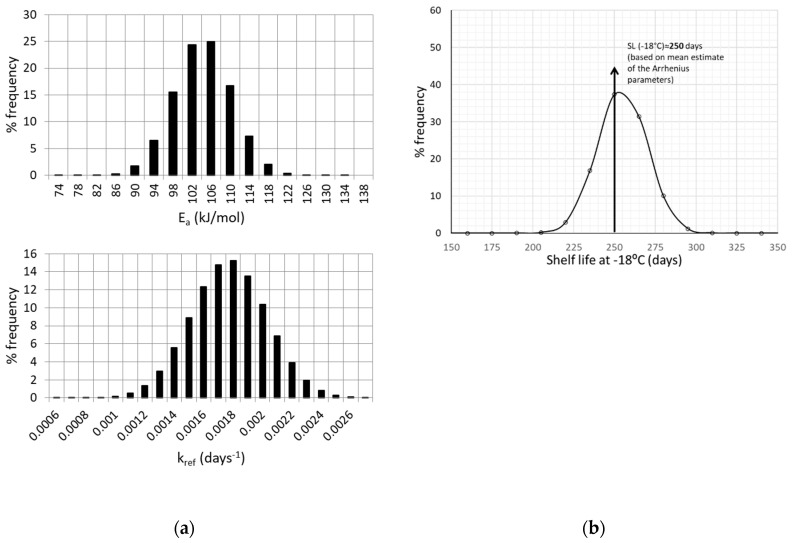
(**a**) Normal distribution of E_a_ and k_ref_ values, based on the mean value and the standard deviation estimated by the one-step non linear analysis and (**b**) Shelf Life estimated at −18 °C.

The next step in the proposed methodology involves a Monte Carlo scheme. applied through a FORTRAN algorithm. This iterative algorithm is applied based on the previous assumption that E_a_ and k_ref_ variability are effectively represented by a normal distribution [14,25,57]. At each iteration, a random number is generated through an appropriate FORTRAN routine function, and a value is assigned to E_a_ and k_ref_ (independently the one from the other). The exact parameter value assigned is based on the discretization of the corresponding normal distribution curve (Figure 2a), and thus the corresponding value frequency. The construction of such Gaussian distributions is based on the estimate of the mean value, and the ±95% C.I. of the kinetic parameters of Equation (6).

Going further with analyzing kinetic data, one cannot overlook the potential correlation between the kinetic parameters (E_a_ and k_ref_), which means that the confidence interval of one parameter depends on the value of the other parameter. Therefore, joint confidence regions were derived using MATLAB and Equation (9); this information was used in order to exclude some of the pairs of values of E_a_-k_ref,_ obtained by the random algorithm of Monte Carlo.

A final step for improving shelf life assessment is based on the incorporation of temperature variability, as depicted in Figure 1, within the Monte Carlo scheme. Applying this nested iterative algorithm by means of a FORTAN code, at each iteration, a double scheme is applied: (1) as already discussed, a random number is generated and a value is assigned to E_a_ and k_ref_ based on the discretization of the corresponding normal distribution curves (2) pairs of E_a_ and k_ref_ that do not fall within the estimated joint confidence intervals are excluded from further analysis and (3) a nested Monte Carlo algorithm is applied, where temperatures at each of the three stages are randomly selected based on the discretization of the distribution curves of Figure 1. With all parameter value assigned, Vitamin C retention is then calculated, based on Equation (6), and the shelf life can be accordingly estimated.

## 3. Results

### 3.1. Application of the Holistic Approach to Shelf Life Prediction in the Frozen Green Peas Cold Chain

In order to test and validate the abovementioned methodology, results from an isothermal study (storage at 5 sub-zero temperatures) of frozen green peas [45] were used and a global 1-step procedure was implemented, using Equation (6). Based on 50% Vitamin C loss, green peas shelf life can be predicted at any arbitrary reference temperature, using Equations (2) and (5). Considering the single value estimates of the Arrhenius parameters, E_a_ = 102.31 ± 17.91 kJ/mol and k_ref_ = 0.00196 ± 0.000795 days^−1^ (as calculated out of a two step procedure and a linear regression analysis), the frozen green peas shelf life is estimated at −20 °C at 390 days (and 248 days at −18 °C). Results from a one step analysis, namely E_a_ = 104.24 ± 11.34 kJ/mol and k_ref_ = 0.00177 ± 0.000494 days^−1^, are slightly different from those estimated via the 2-step analysis. Using a one step analysis, frozen green peas shelf life is also estimated at −18 °C at approximately 250 days. It can be also observed that when applying the two-step approach, the 95% C.I., (calculated via regression analysis) are usually wider than those calculated with a global-one step approach [22].

As discussed earlier, the next step involves the application of a Monte Carlo simulation scheme, assuming that E_a_ and k_ref_ variabilities are described by a normal distribution (Figure 2a). The Gaussian distributions illustrated in Figure 2a were constructed based on the estimate of the mean value, (for example E_a_ = 104.24 kJ/mol) and its standard deviation, (in the case of E_a_, σ = 5.8 kJ/mol). The same procedure was followed to construct the corresponding distribution curve for k_ref_.

The Monte Carlo algorithm aimed at ascribing specific values to the E_a_ and k_ref_ parameters, based on their normal distribution curve, in order to estimate the shelf life (Equation (6)) at an arbitrarily chosen temperature of −18 °C, using the 50% Vitamin C loss as the acceptability limit. Results for Shelf Life (SL) calculation, including its uncertainty, are depicted in Figure 2b, expressed as a frequency curve with a mean value (SL estimate) ± 95% C.I., equal to 254.2 ± 29.9 days, giving a more realistic prediction than the single value estimation of 250 days, based on Arrhenius parameters’ mean estimated values. The necessary number of Monte Carlo simulations for a specific kinetic model is not clearly defined in literature (ranging from some hundreds to few thousands), since there are other factors, such as the degrees of freedom, the range of inputs, possible parameter interactions, etc., that affect algorithm performance [58]. However, the number of 10^4^ (applied in this study) seems to be a frequently used number of iterations [59,60] and an acceptable compromise between computing power/time and result accuracy.

The aforementioned procedure is in agreement with the approach described in [29], where the confidence intervals of E_a_ and k_ref_ parameters of the Arrhenius equation were estimated for degradation of cyanidins under dynamic conditions, by generating artificial data of the initial measurements, superimposing the experimental error. The use of a Gaussian distribution is a common practice in food engineering [18,61]; however, the same methodology can be implemented in case a different probability distribution describes better data variability [28,62,63,64,65,66].

**Figure 3:**
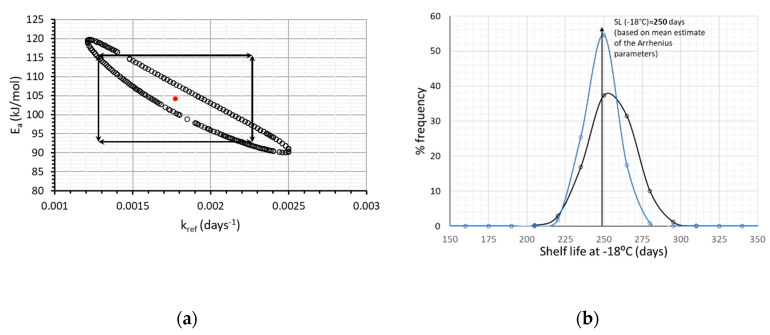
(**a**) Joint Confidence Intervals, depicting the correlation between parameters E_a_ and k_ref_ and (**b**) Shelf Life estimated at −18 °C, taking into account the correlation of the two kinetic parameters (). Black line represents the SL distribution without considering E_a_-k_ref_ correlation, blue line after excluding the E_a_-k_ref_ pairs of Figure 3a.

As a next step, the joint confidence intervals estimated by MATLAB and Equation (9) (Figure 3a), were also considered and, consequently, some pairs of E_a_-k_ref,_ values obtained by the random algorithm of Monte Carlo, were excluded. Given these pairs of E_a_ and k_ref_, the shelf life at −18 °C (arbitrarily chosen) is calculated, which is described by a distribution even narrower than the one of Figure 2b. (Figure 3b, blue line), deriving a shelf life of 248.5 ± 21.3 days (95% C.I.).

**Figure 4:**
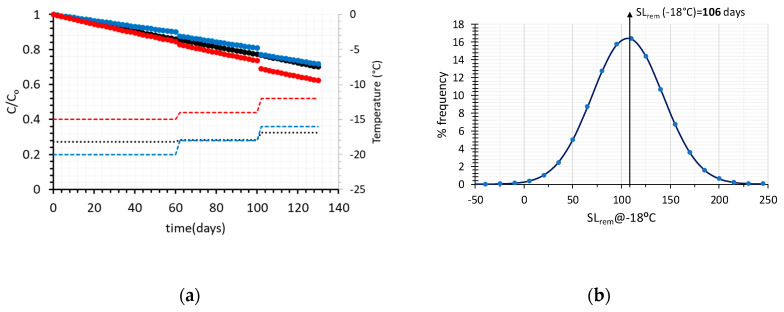
(**a**) Vitamin C degradation, for different temperature scenarios of the cold chain of frozen green peas (black line represent the C/Co value for the average temperatures of all stages) and (**b**) Remaining Shelf Life estimated at a reference temperature of −18 °C, for the 2000 different distribution scenarios through Monte Carlo technique (kinetic parameters E_a_-k_ref_ in Equation (6) assumed fixed).

In order to implement this methodology in the real cold chain, and make an attempt to expand it by introducing the recorded temperature variability, a 130-days distribution scenario of frozen green peas is assumed, including 60 days at the warehouse (Figure 1a), 40 days at the retail level (Figure 1b) and 30 days at the domestic freezer (Figure 1c). The main goal is to predict the remaining shelf life of such a product prior to consumption, taking into account not only the estimated uncertainty of kinetic parameters, but also temperature variability at each stage of the cold chain (Figure 1). When only average temperatures are introduced in Equation (6), and mean estimates of kinetic parameters E_a_-k_ref_ are considered, the remaining Shelf Life after 130 days of handling is estimated at 106 days (at a ‘reference’ temperature of −18 °C), (black line, Figure 4a).

In Figure 4a, the degradation of Vitamin C is depicted vs. time, for different handling scenarios, throughout the frozen green peas cold chain, based on temperature data retrieved from Figure 1a–c. Applying a Monte Carlo iterative scheme, 10^4^ different temperature cases (3 of the random scenarios are depicted in Figure 4a, using different colours) were ran and the remaining shelf life at the end of the 130 days cycle was calculated (Figure 4b). In this approach, it is important to bear in mind that kinetic parameters E_a_-k_ref_ in Equation (6) were assumed constant, with fixed values equal to the mean estimates of regression analysis (Figure 2a).

**Figure 5:**
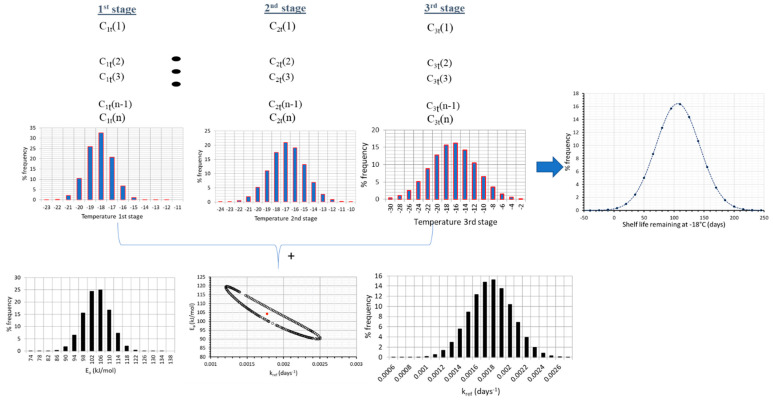
Flow chart of the methodology used, showing the remaining shelf life of frozen green peas, after 130 days in the cold chain, when both temperature variability and parameter uncertainty were incorporated within model’s prediction algorithm.

**Figure 6:**
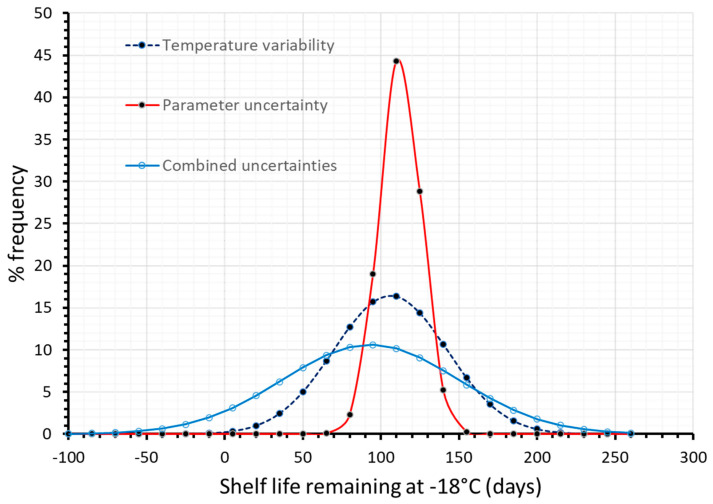
Remaining shelf life of frozen green peas, after 130 days in the cold chain, when only temperature variability (dashed blue line), only parameter uncertainty (red line) or both temperature variability and parameter uncertainty (solid light blue line) were incorporated within model’s prediction algorithm.

Finally, in a further attempt to improve quality change predictions, and therefore obtain more realistic estimations of the remaining shelf life, kinetic parameter uncertainty was also incorporated in a double Monte Carlo scheme throughout the 130 days cycle (Figure 5), and thus all sources of ‘error’ (parameter uncertainty and temperature variability) were integrated in a holistic approach of kinetic data analysis. In this case, temperature variability is taken into account (by a Monte Carlo iterative algorithm using the temperature distributions of Figure 1a–c), and a second, ‘nested’ Monte Carlo scheme is used during each of the three distinct stages where a random pair of E_a_-k_ref_ is selected considering not merely the distributions depicted in Figure 2a, but also the intercorrelation between these parameters, as depicted in the Joint Confidence Intervals (Figure 3a). Having incorporated within the model both temperature variability and parameter uncertainty, remaining shelf life predictions become significantly broader, as it is clearly depicted in Figure 6, where results deriving from different approaches are comparatively depicted. When all possible sources of error are incorporated, remaining shelf life is estimated at 93.4 ± 110.5 (days), vs. 106.0 ± 71.3 (days) when only temperature variability is considered, vs. 112.4 ± 26.0 when temperatures are assumed constant during the three stages (and equal to the mean estimate of each distribution of Figure 1), accounting only for parameter uncertainty. All these predictions can also be compared to the mean value predicted (106 days) without considering any source of error.

### 3.2. Effect of Parameter Uncertainty

**Figure 7:**
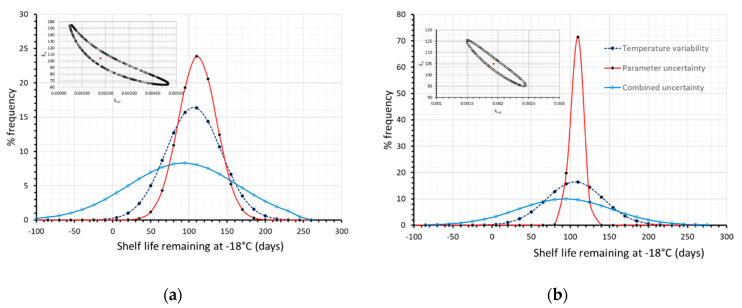
Remaining shelf life of frozen green peas, after 130 days in the cold chain, when only temperature variability (dashed blue line), only parameter uncertainty (red line) or both temperature variability and parameter uncertainty (solid light blue line) were incorporated within model’s prediction algorithm. (**a**) when assuming broader 95% C.I. (**b**) when assuming narrower 95% C.I. Relative Joint Confidence Regions are depicted within plots.

In this case, it can also be observed that the prevailing source of error is mainly attributed to the temperature variability, and less to the kinetic parameter uncertainty; this could be justified by the relatively narrow ±95% confidence intervals of the kinetic parameters, E_a_-k_ref_, as obtained by the 1-step non linear regression, based on the Vitamin C measurements. If an artificial error is introduced in raw data, and the derived ±95% confidence intervals (using MATLAB) are assumed broader (as depicted in the joint confidence region within Figure 7a), it can be observed that the contribution of parameter uncertainty becomes more pronounced, than that depicted in Figure 6. Similarly, if initial measurements are improved so as to provide much narrower ±95% confidence intervals (as depicted in the joint confidence region within Figure 7b), one could conclude that parameter uncertainty introduces a relatively small error in comparison to the effect of the storage temperature variability that could practically be neglected.

### 3.3. Effect of Temperature Variability

**Figure 8:**
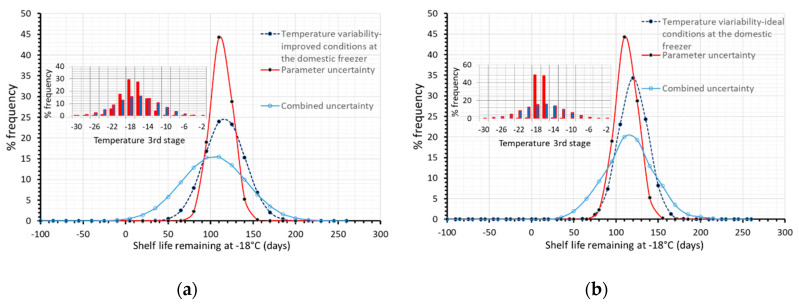
Remaining shelf life of frozen green peas, after 130 days in the cold chain, when only temperature variability (dashed blue line), only parameter uncertainty (red line) or both temperature variability and parameter uncertainty (solid light blue line) were incorporated within model’s prediction algorithm. (**a**) for slightly improved temperature conditions at the domestic level (**b**) assuming ideal conditions at the domestic level. Temperature conditions of the third stage are depicted within plots.

Based on our results, temperature conditions throughout the current cold chain, deviating from the ideal ones, have an important impact on overall uncertainty, when assessing the quality status at any point of distribution. A ‘what if’ analysis was also performed regarding temperature variability (Figure 8), assuming a narrower distribution (red, narrower distribution within both Figure 8a,b), during storage at the domestic freezer (3rd stage), which is found to be the weakest link of the chain. As it can be seen in Figure 8, the remaining shelf life is estimated to be 103.9 ± 74.8 days (Figure 8a, narrower temperature conditions during home storage), and 113.1 ± 65.6 days (Figure 8b, assuming reference conditions during home storage), a quite narrower, improved distribution compared to the 93.4 ± 110.5 days, derived when temperatures during the third stage followed the broad, often abusive distribution of Figure 1c (based on real data).

## 4. Conclusions

When revisiting recent literature on kinetics of food-related reactions, limited number of investigations have studied the significance of statistical aspects, such as the effect of kinetic parameter uncertainty. Additionally, few researches take into account quantitatively the significant temperature variability, recorded in all stages of the actual cold chain of frozen foods. Most physicochemical properties of raw food materials, even of the same species or cultivar, are found to assume values in a wide range, a variability that is difficult to be quantified and properly introduced within kinetic models. Therefore, next to the inconsistency of the initial quality, it is important to investigate in depth the significance and the impact of parameters uncertainty (as expressed by their 95% Confidence Intervals), as well as temperature conditions’ variability, especially when the goal is to derive realistic model predictions. Reporting of parameter uncertainty is essential when kinetic results are presented. The use of computational tools such as Monte Carlo technique can provide a practical approach in providing an integrated picture of the impact of both experimental and statistical uncertainties as well as the potential conditions’ dynamics.

In this work, by applying the proposed technique with the double Monte Carlo scheme on Vitamin C degradation of frozen green peas, a broader, more realistic prediction of the remaining shelf life was obtained based on the fact that the model incorporates more realistic conditions, as model validation was not implemented in this study. The aforementioned methodology allows also for a sensitivity analysis, revealing the importance of reducing the 95% Confidence Intervals of the kinetic parameters (possibly by optimizing the experimental design), and/or by improving temperature conditions of the cold chain, actions that would lead to narrower distributions of the remaining shelf life, with less unacceptable products.

As a final remark, it should be pointed out that, although the preceding analysis was exemplified by application on a first-order reaction (Vitamin C loss) and temperature dependence modelled by the Arrhenius equation, the same approach, using identical steps of methodology, can be implemented when an alternative primary or secondary model is used to describe kinetic data.
